# Is marriage ‘just a paper’? Why men and women choose cohabitation over marriage in the Philippines: insights from focus group data

**DOI:** 10.1186/s41118-025-00263-2

**Published:** 2025-08-27

**Authors:** Bernice Kuang

**Affiliations:** Department of Social Statistics & Demography, Faculty of Social, Human & Mathematical Sciences, University of Southampton, Highfield Campus, Southampton, SO17 1BJ UK

## Introduction

Cohabitating families are often associated with individualistic cultures and family systems, in contrast to family-centric cultures which are characterized by pro-marriage, pro-natalist norms, and strong adherence to family expectations, tradition, and religious and sexual mores (Lesthaeghe, 2010). The Philippines has a strong family-centric culture and conservative family policies—divorce and abortion are illegal and family planning access is limited—and the majority of the population is Roman Catholic (Jocson, 2020; Medina, 2015; Morillo et al., 2013). Surprisingly, cohabitation and childbearing within cohabitation have increased rapidly in the Philippines, with more than half of first births now occurring outside of marriage, mostly to cohabiting women (Kuang et al. 2017; Kuang et al. 2019). However, little is known about why people have started to choose cohabitation versus marriage.

I use focus groups to investigate what reasons lead people to cohabit or marry and what are the perceived meanings, advantages, and disadvantages of cohabitation and marriage. Examining why people cohabit or marry may illuminate whether their decisions are more motivated by individualistic or family-centric tendencies, which is of particular theoretical interest because the Philippines is generally viewed as a family-centric society while cohabitation is viewed as an individualistic family form. In individualized societies, cohabitation and marriage have been compared using individualistic lenses to understand why people choose cohabitation or marriage and to what extent their functions are similar (Hiekel & Keizer, 2015; Kiernan, 2001). Incorporating a family-centric perspective to study the rise of cohabitation in an unexpected context like the Philippines sheds new insights about cohabitation as a social phenomenon.

Quantitative studies demonstrate the Philippines’ rising cohabitation and nonmarital fertility (Abalos, 2023, Kuang et al. 2019), but qualitative studies can explore the meaning of these practices and more fully describe them. Qualitative data can also examine how changing family behaviors relate to shifting social norms and are not constrained by predetermined categories, questions, and responses as survey data are (Perelli-Harris & Bernardi, 2015). Previous Philippine studies revealed gaps between reported attitudes and relationship behaviors (Kabamalan, 2004; Williams et al., 2007; Xenos & Kabamalan, 2007), which qualitative data may also help to elucidate. Finally, existing qualitative studies of cohabitation tend to focus on Western contexts, to the exclusion of family-centric Asian countries, even as cohabitation has increased worldwide. The Philippines is a country of over 109 million people (PSA 2021), with one of the largest diaspora populations in the world, who nonetheless retain very strong ties with their origin country (Nicolas, 2016, Roldan 2024). This paper not only diversifies existing perspectives, building on the Western-dominated debate, it also investigates a population with worldwide relevance that has to date received little attention in the international demographic literature.

## Background

### Individualism and relationships

In an individualistic culture, people are not beholden to class, gender, religious, or family expectations, but focus instead on personal needs. For intimate relationships, the individual need for romantic love, emotional satisfaction, and self-fulfillment may precede long-term commitment or feelings of relationship obligation, and relationship decisions are shaped by the individual or couple, and not by family, state, or religious expectations (Beck & Beck-Gernsheim, 1995; Giddens, 1992). In this way, the life course de-standardizes into “biographies of individual choice” (Beck, 1992). Increased cohabitation and legal recognition of cohabiting families are commonly noted as evidence of individualization (Lesthaeghe, 2010) and cohabitation is often discussed from the individualism perspective (Berghammer et al., 2014; Hiekel & Keizer, 2015).

In individualized cultures where cohabiting families are accepted, cohabitation and marriage may have similar functions as family forms (Kiernan, 2001) and in some cases, cohabiting couples may be viewed as committed to each other as married couples (Berrington et al. 2015), raising the question of why people choose one arrangement over the other. People may cohabit if they view it as less fraught with gendered and social expectations compared with marriage, which may particularly appeal to women (Edin & Kefalas, 2005; Reed, 2006), whereas marriage could be a personal risk reduction strategy in case of union dissolution, providing economic or legal protection, alimony or child support, and ensuring property rights, to an extent that cohabitation does not (Hiekel & Keizer, 2015; Perelli-Harris et al., 2017). And although cohabitation may have the potential to be as serious as marriage, people could also cohabit to allow themselves more freedom (Berghammer et al., 2014), with less personal commitment (Duncan et al., 2005; Berrington et al. 2015). As unions increasingly begin with cohabitation, people no longer marry to mark the beginning of a relationship but instead to publicly confirm an existing relationship, shifting the meaning of marriage away from its institutional origins toward an emotional, symbolic significance (Cherlin, 2004; Kiernan, 2004). For cohabiters with children, getting married may not change daily life but may change the broader societal perception of the couple, and the couple’s self-perceptions and standards of behavior, love, and commitment (Reed, 2006). Additionally, if marriage has shifted away from its traditional social function, people may choose to marry or cohabit simply as personal preferences and not as ideological statements.

### Familism and relationships

Familism refers to the notion that the collective needs of the family are a higher priority than the needs of individuals (Cardoso & Thompson, 2010; Cauce & Domenech-Rodriguez, 2002). Key components of familism are perceived support, emotional closeness, family obligation, and adherence to family expectations (Choi et al., 2021; Sabogal et al., 1987). The family unit is cohesive, conformist, and interdependent (Cardoso & Thompson, 2010), and there is strong adherence to tradition, religious mores, and a rigid ordering of life course events (Lesthaeghe, 2010). The prioritization of family harmony and expectations contrasts with individualism, and cohabiting families are not commonly associated with family-centric cultures. Instead, cohabitation and nonmarital fertility usually represent deviation from traditional values and religious mores. Even where stigma is minimal, cohabitation may still be viewed less favorably than marriage, with marriage seen as a more enduring and fulfilling romantic relationship, recognized by law and society (Perelli-Harris et al., 2014; Reed, 2006). Marriage may be perceived to entail higher levels of moral obligation between partners to stay in the relationship, referred to as moral commitment, and may also benefit from greater structural commitment, such as the social and financial ties that bind a married couple together, and the stigma, legal, and financial costs of divorce (Berrington et al. 2015). Overall, marriage is more consistent with the standard life course and traditional values of family-centric cultures.

Nonetheless, people may still cohabit for family-oriented reasons. This has been observed in populations where childbearing in cohabitation is common even while marriage is highly valued, such as among disadvantaged groups in the United States (Edin & Kefalas, 2005). In this context, cohabitants value marriage and wish to marry but feel they need to reach a certain economic standard (have money for a wedding, own a home, attain financial security) before marrying. Large-scale uptake of cohabitation may also be associated with a ‘pattern of disadvantage’ and ‘diverging destinies’, which argues that the advantaged follow one path toward cohabitation and then stable marriage, while the disadvantaged are more likely to have children outside of marriage or within unstable partnerships (McLanahan, 2004; Perelli-Harris et al., 2010), even while marriage remains valued overall. On the other hand, Sassler and Miller (2022) argue that middle-class individuals cohabit without the precise aim of marriage and see cohabitation as part of the development of a relationship that can potentially intensify and lead to marriage, while people from more disadvantaged backgrounds view cohabitation as equal to marriage.

Childbearing in cohabitation is also increasingly common across socioeconomic groups in many Latin American countries (Laplante 2015) despite the family-centric nature of Hispanic cultures (Sabogal 1987). For instance, cohabitation may be a response to a nonmarital pregnancy or birth and not an ideological rejection of marriage, allowing a couple to co-parent without prematurely committing to an untested or substandard relationship or if the couple feel they have not achieved the economic standard perceived as a prerequisite for marriage (Edin & Kefalas, 2005; Reed, 2006). Cohabitation may also be a strategy to cope with the economic uncertainty of unemployment and precarious work common in global labor markets because cohabitation is viewed as more reversible and temporary than marriage and thus more suitable for an uncertain life (Perelli-Harris et al., 2010).

In such cases, the normative role of parenthood is strong enough to bring a couple together to cohabit, but not necessarily to marry if the relationship is substandard (Reed, 2006). Prioritizing a two-parent family and ensuring relationship quality before marriage preserves the importance of family and the value of marriage. Cohabitation decisions may also be child-centric, such as when single parents consider introducing a stepparent into their children’s lives (Reid & Golub, 2015). In family-centric cultures such as Japan, China, and Thailand, where cohabitation has become more common, cohabitation is typically a precursor to marriage and marriage remains the clear goal (Lesthaeghe, 2010; Raymo et al., 2015; Yu & Xie, 2021). Moreover, although premarital pregnancy has become increasingly common, especially among cohabiters, nonmarital fertility remains extremely taboo. In Taiwan, cohabitation has increased over time and has evolved toward becoming an alternative to dating—a short-term arrangement where marriage is not the objective; however, cohabitation is still not seen as an appropriate context for having children (Cheng, 2023). In these countries, the sequence of the life course remains strict.

### Familism in the Philippines

Philippine culture emphasizes familial closeness and obligation (Jocson, 2020; Medina, 2015; Morillo et al., 2013). Family clans historically worked and lived together, relying on kin networks for social, legal, political, and economic structures. To this day, there is a strong reliance on family ties (Francia 2010; Jocson, 2020), including family-based social support systems (Alesina & Giuliano, 2013). Reciprocity is important to reaffirm kinship ties, and supporting relatives is expected (Miralao, 1997; Morillo et al., 2013). With the rise of labour migration, such expectations remain strong, and families function across international networks, as evidenced by a strong culture of remittances (Go, 1993). Familism is strongly associated with many Asian countries, transcending religion, traditional customs, and philosophy (Jeung et al., 2019; Raymo et al., 2015) and is also a cornerstone of Hispanic culture (Sabogal 1987; Vega, 1990; Hartnett & Parrado, 2012), both of which have cultural relevance in the Philippines as an Asian country and former Spanish colony.

In the Philippines, marriage traditionally served family-centric purposes as an alliance between clans. Courtship and marriage alliances were traditionally managed by kin, with parents exercising a large degree of control over their children’s relationship decisions (Medina, 2015). Over time, the emphasis on marriage as a familial duty has waned (Williams & Guest, 2005) and traditional courtship has given way to more modern forms of dating that are less supervised and family-centric. People became more likely to exercise personal choice in mate selection and see marriage as important for practical and personal reasons, such as for company, to have children for old age support, and to provide structure and discipline to one’s life (Williams & Guest, 2005).

The importance of family reciprocity is linked to the Philippines’ preference for large families and child-centric culture (Medina, 2015; Miralao, 1997; Morillo et al., 2013). Children are expected to assist their parents—such as with housework, childcare, financial contributions, and old age care (Medina, 2015). Children also play an important family role by linking maternal and paternal kin groups to form the bilaterally extended family, effectively expanding the group from which resources and support may be expected (Medina, 2015). Finally, children are also perceived to provide key emotional benefits such as companionship, love, happiness, and a sense of purpose (Bulatao, 1978). Although family behaviors and structures have shifted to incorporate less traditional modalities (Medina, 2015), children remain highly valued and two-parent households are still viewed as normative and essential for child well-being (Morillo et al., 2013). Furthermore, the family is still perceived to be defined by emotional closeness, support, and warmth (Tarroja, 2010), highlighting the persistent importance of family in the Philippines.

### Cohabitation context in the Philippines

Older studies have argued that cohabitation was not seen as an accepted substitute for marriage and was widely disapproved of, even among young people who were most likely to cohabit (Kabamalan, 2004; Medina, 2015; Williams et al., 2007). Nonetheless, approximately 19 percent of 15- to 49-year-old women in the Philippines are currently cohabiting, including one third of 25–29 year olds (PSA and ICF, 2023) and there has been a consistent increase over time (Kuang et al. 2019; Abalos, 2023). Because the Philippines is the only country in the world where divorce is illegal for most of the population, cohabitation may be a way to test a relationship before marriage (Williams & Guest, 2005) or a strategy to avoid marriage altogether. Cohabitation is also the only arrangement available to married people who are estranged from their spouses and wish to re-partner but cannot legally remarry. The prevalence of legal separation is very low in the Philippines, due to the complexity of the procedure, strong kinship ties, stigma, and the preference for two-parent households (Abalos, 2017; Morillo et al., 2013). The only way to legally dissolve a marriage is annulment, which is costly and complex. Nonetheless, petitions for annulment have more than doubled between 2001 and 2014 (Abalos, 2017), alongside a decline in the number of registered marriages (PSA 2017). Increasing public support for legalizing divorce suggests that social norms may be liberalizing (Miller 2008; Laranas 2016) although support is still nowhere near universal (Abalos, 2017).

## Data and methods

### Motivation for using focus groups

The interactive group discussion method is useful for exploring norms and values that govern behaviors because values and norms are not fixed traits that occur in isolation but are instead replicated, adjusted, or produced through social interactions (Liamputtong, 2011; Stewart et al., 2007). In social interactions, people experience acceptance or rejection of a given behavior, which further transmits and reinforces shared beliefs and attitudes; social interactions are shaped by compliance with norms and, in turn, norms are reaffirmed by social interactions (Perelli-Harris & Bernardi, 2015). Such norms may relate to the proscription or prescription of behaviors, and the discussion format documents the arguments for and against them. Unlike in-depth interviews, focus groups create a situation where people can discuss and challenge each other (Wilkinson 2003; Barbour, 2007). Lastly, the use of focus groups is particularly useful for explaining family change because ideational change theories highlight the importance of normative context in shaping new behaviors (Thornton 2001; van de Kaa 2001; Lesthaeghe, 2010).

### Focus group discussions

Together with a team from the University of the Philippines Population Institute, I held 8 focus groups in 2016 assessing how cohabiting and married men and women viewed cohabitation and marriage. Moderators asked respondents their opinions on why some people cohabit and why some people marry, why they thought cohabitation was becoming more popular, and whether they thought cohabitation was now accepted by society. Moderators also asked whether having a child was a good reason for a couple to marry or if there was a certain time period after which a cohabiting couple should marry. Probes were provided for each question to stimulate conversation if necessary. Most discussions had 6–8 participants, for a total of 54 respondents, 26 women and 28 men. Only women and men over 18 were recruited. Discussions were held with women and men aged 22–62 from one urban neighborhood, or *barangay* (Quezon City, National Capital Region) and one rural *barangay* (Hermosa, Bataan province). Focus group data cannot be used to draw conclusions about subgroup differences because the nature of such qualitative data is that it cannot represent an entire subgroup. Instead, groups were divided by sex, residence, and union status to encourage open discussion and stimulate conversation so people could feel free to express opinions on potentially sensitive subjects such as premarital sex, nonmarital childbearing, cohabitation, and divorce. By stratifying people based on similar background, discussions were among people who were more likely to have similar views (Barbour 2010), coalescing around a more homogenous set of norms and themes. Table 1 presents the background information on respondents in each focus group.

**Table 1:**
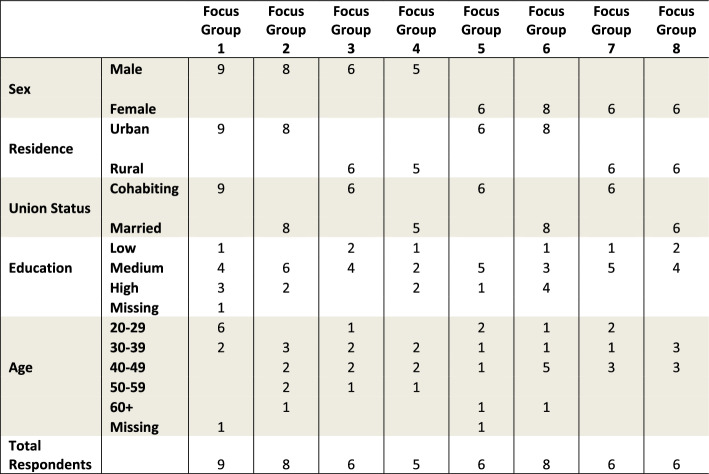
Background characteristics of focus group respondents in each focus group

With the wide range of respondent ages, moderators made a deliberate effort to encourage everyone to participate in the conversation, in case either the oldest or youngest participants felt hesitant to share views different from the majority. In cases where respondents were asked about issues pertaining to a life stage they were no longer in or had not yet reached (i.e., how to advise adult children on relationships), the moderator encouraged respondents to reflect hypothetically on the question and share their views. Respondents all came from the same small community, and most had at least a high school education, which limited the range of represented socioeconomic statuses to the exclusion of very poor or very affluent people, as well as both the very low and very highly educated. Most participants had a medium level of education, which I defined as having high school education, although about a fifth of respondents had university education. The focus groups with cohabiters tended to have younger participants than the focus groups with legally married participants, and the urban focus groups tended to have participants with higher education, consistent with the broad national patterns. The vast majority of respondents, 78 percent, reported that they had never been in a previous union (i.e., that the union they were currently in was their first union). Those who did report being in a previous union were all cohabiters, four of whom reported that their previous union was a legal marriage.

Although I held discussions among both urban and rural residents, the rural residents lived only a few hours’ drive from the National Capital Region, and were not by any means the most isolated rural dwellers in the Philippines. The vast majority of respondents were parents and due to the recruitment method, everyone reported being in either a legal marriage or cohabiting relationship, which may also be reflected in people’s views of childbearing and partnership. Finally, in group discussions, sometimes the most outspoken people are the most likely to share their opinions and dominate the conversation. Moderators counterbalanced this potential drawback by directly inviting less vocal people to share their views.

### Data analysis

All discussions were audio recorded and lasted between 60 to 90 min. All audio recordings were transcribed verbatim into Filipino and translated into English by two members of the research team to ensure as much consistency as possible. I coded transcripts using the qualitative data analysis software NVivo 13 with a thematic coding process using themes derived from theoretical explanations for cohabitation versus marriage from existing literature, such as economic uncertainty, commitment, and family expectations. With these themes, I analyzed the data using a top-down, deductive approach, coding the data by theme and identifying the most recurrent findings and the quotations that best reflected them. In order not to be limited by existing theories and explanations, I also considered new perspectives in a bottom-up, inductive approach. This involved identifying themes raised by respondents outside of the range of expected theoretical explanations from the literature, most of which is from a Western context—for example, the high valuation of children and the role of surnames and lineage in relationship decisions. Because of the unique context of the Philippines even within the Southeast Asian region, it was important to apply a method to induce themes that did not lean on existing theories in order to challenge what we think we know about the meaning of cohabitation as a family form. Finally, I categorized the findings to determine whether reasons were more consistent with individualism or familism, which is explained in “Discussion” section.

## Results

### Children and relationship decisions

Although the objective of the focus groups was to explore cohabitation and marriage, respondents independently raised the issue of children. The consistent mention of children was a revealing component of the data, reflecting the child-centric nature of Philippine culture and in stark contrast to similar data collected in European countries, which focused on the couple (Perelli-Harris & Bernardi, 2015). This section presents findings that demonstrate how respondents consistently discussed why one would cohabit or marry from a child-centric perspective. Results in subsequent sections, which for example focus on economic reasons, are also presented without disentangling mention of children and family.

Respondents often shared their views on the benefits and disadvantages of cohabitation and marriage based on how their children would be affected. Some respondents directly associated relationships with having children, without acknowledging the possibility of delaying or not having children within a relationship. For example, respondents were asked whether a person needed to marry to feel complete, to explore how people viewed marriage as a path to self-fulfillment. Male respondents reported that marriage and the inevitable transition to family life motivated them to change destructive behaviors, giving their lives purpose and stability. Because marriage implicitly meant having children, men reported the same benefits for both.

Respondent: “Before, I don’t [didn’t] really want to get married. … And then, I will see a family walking in the streets… They are complete and happy. But me? I am walking alone… [Now] I have four children. I am already complete… You are always worrying but it is very fulfilling.”

-rural married male, age 54, college educated.

Women also spoke of partnering as synonymous with childbearing, for example, by explaining that they did not want their daughters to have boyfriends until they were ready to have children. In contrast to men, women were more likely to evaluate fulfillment from marriage and fulfillment from childbearing separately. Having children was reportedly a universal requirement for happiness, but having a husband or partner was neither necessary nor sufficient for happiness, suggesting less alignment with traditional values in the context of relationships and more emphasis on individual partnership preferences, despite child-centric tendencies.

Moderator: “But who do you think are happier, those who have spouses or those who have none?”

Respondent 1: “It depends on the situation eh.”

Respondent 2: “It depends on the person… On where she’ll find happiness… joy.”

-rural married females, ages 30–40, high school educated.

Respondent: “Your world revolves around your children- maybe a husband is secondary. As long as you have a child, life is complete”.

-urban cohabiting female, age 30, high school educated.

### Reasons to cohabit or marry

Both married and cohabiting respondents reported that cohabitation was previously stigmatized but had now become acceptable, both as a prelude to marriage and as a longer term arrangement for having children. Nonetheless, respondents said marriage was important and had benefits cohabitation lacked, and they were neither opposed to marriage nor of the mind that it was essential for everyone to marry. Female respondents in particular noted that cohabitation norms had changed because sexual mores were more relaxed, and people were “liberated”, “modern”, and “practical” while men tended to disagree on whether this was a positive development. When asked why people now cohabit instead of marrying, respondents gave a wide range of reasons, reflecting practical adaptations to circumstances, emotional reasons, and changing social expectations. In the next sections, I present respondents’ views on why people cohabit and what the practical, emotional, social benefits are, as well as how cohabitation compares with marriage.

### Practical reasons

#### Economic concerns

Financial concerns were one of the most consistently mentioned reasons to cohabit instead of marry. Respondents across focus groups said cohabiting was more financially practical than marrying and hosting the expected church wedding. By cohabiting, couples can buy themselves time to save for a wedding, avoid the expense of a wedding altogether, or allocate resources toward their children instead. However, respondents were well aware of more affordable options to marry—at city hall or in a mass wedding—suggesting there are more than financial concerns alone. Instead, a lack of urgency or priority to marry may also be at play. Indeed, several cohabiting subjects who said they planned to marry also admitted that paying for a wedding was not a priority, especially given the competing needs of their children, and cohabitation was viewed as a strategy to save resources, which would not necessarily be ultimately allocated to a wedding.

Moderator: “For example right now, I will give each of you five thousand [pesos].^1^ In your opinion, will you get married?”.

Respondent 1: “Ay, not anymore, Ma'am. I will give it to my children…”

Respondent 2: “And also, I would also invest for my kids.”

-rural cohabiting females, age 40–44, high school educated.

Another rural cohabiting male respondent replied that he had not married his partner because they were “always busy”, highlighting the low priority of marriage. Some explained that unmarried couples with children were simply not “excited” to marry and were “comfortable” after many years together raising children, again emphasizing the lack of pressure to marry.

### Policy changes

Demonstrating causal policy effects cannot be done with cross-sectional data. My aim is not to ascertain causality but to examine social relationship norms and how cohabitation is viewed based on respondent perception. Respondents believed policy changes—specifically, changes to the Family Code (1987)—were key to the increased appeal and normalization of cohabitation, leading more people to cohabit. Discussion focused on policies impacting children, again highlighting respondents’ child-centric priorities. Respondents believed the most crucial policy change that caused people to start cohabiting more was an amendment allowing children of unmarried parents to use their father’s surname instead of their mother’s because a child having their father’s surname was viewed as extremely important, signaling family membership and paternal acknowledgment, and seen as a key benefit of marriage (Williams & Guest, 2005). Respondents also said that another reason more people cohabit was because children could now claim parental benefits and register for school even if their parents were unmarried. In addition to incentivizing cohabitation and dis-incentivizing marriage, respondents reported that these policy changes improved the acceptance and uptake of cohabitation. Nevertheless, respondents still believed marriage provided more comprehensive legal support for both partners and children, such as access to spousal benefits, spousal pensions, and social security. Although respondents believed that children of cohabiters could avail themselves of many benefits, they still believed having a legally documented marriage would make administrative tasks simpler for their children.

### Cohabitation as relationship insurance

Another reason to cohabit was to test the relationship. Respondents saw cohabitation as useful for partners to get to know each other and “check” the relationship. Female respondents in particular advised that it was not wise to rush into marriage and that a couple should take time to get to know each other in cohabitation. When asked how long cohabiters could live together before they should get married, some respondents suggested one to five years while others said there was no set timeframe. Even a couple with multiple children together could “still be in the process of getting to know each other.” While some cohabiters reported a desire to marry their partners, others did not have a clear aim to marry. This indicates that cohabitation may not always be viewed as a precursor to marriage as previous Philippine studies have asserted but may also be a “testing ground”, or indeed even an alternative to dating (Cheng, 2023; Perelli-Harris et al., 2014).

Moderator: “When is the right time [to marry] for you? What are the signs that it’s already the right time?”

Respondent 1: “It’s not yet right for me”

Respondent 2: “For me, when the time comes that I want to be tied to another person already, but for now, not yet”.

-rural cohabiting males, ages 38–41, high school educated.

Respondents also viewed cohabitation as a way to avoid the possibility of being trapped in an unhappy marriage, given the lack of access to divorce, either by using cohabitation as a test period to lower the chance of future problems or as a strategy for avoiding marriage altogether. One respondent summed up many of these themes when discussing his own experiences. Notably, this respondent uses the term “wife” even though he is not legally married to his partner. Throughout the focus groups, cohabiters often referred to their partners as their spouses, even though they were not legally married.

Respondent: “For us, (we) live in first, because my wife is thinking about what happened to her mother and father who got married then got separated. So that became her thing, her fear. So we talked about living in first. Because in terms of financial problem, to get married, because there are mass weddings anyway, which are free. It's only nowadays that people make a problem about weddings because they make it grand. Getting married does not have to be grand. It just needs to be in the eyes of God, even if it's free that's okay… judge or mass wedding, that's okay. For us, we lived in first because she fears getting married then getting separated. And we're also strengthening our relationship in that we're trying this live in status first before we get married.”

-urban cohabiting male, age 21, college educated.

### Emotional reasons

#### Love and personal commitment in cohabitation

We asked respondents if they believed people choose to cohabit because of a lower level of love, commitment, and obligation, compared with marriage. Respondents believed that people married for love, relationship security, and their children’s future, but believed that cohabitation could also have love and some degree of security. Several respondents said cohabiting relationships could be just as personally committed and stable as marriage, reasoning that to some, being married was “just a paper” and a good relationship “does not depend on marriage” but on the couple and their specific relationship and circumstances. Nonetheless, respondents admitted that they may prefer for their children to legally marry, though in reality, many of their adult children had children and were cohabiters, suggesting that although there is tolerance of different life courses, some may still be idealized.

Respondents also repeatedly reported that a legal marriage did not ensure relationship success. Across focus groups, respondents independently raised the point that not all marriages were happy or long lasting, whereas some cohabiting relationships were. Several respondents gave examples of long-term cohabiters who separated soon after marrying, suggesting marriage does not inherently make a relationship happier or more personally committed. One rural female cohabiter reported that her parents married after 43 years of cohabitation and separated shortly thereafter. She said *“it is horrible if people enter into unions and get married, but they would only break up in the end. They have been together for so long and yet did not marry. However, when they were finally married, they ended up separating.”*

### Moral commitment

Although respondents said a cohabiting relationship could be as stable, loving, and personally committed as a marriage, some respondents, especially male respondents, also believed that marriage usually had higher levels of expected moral commitment, although adherence to this expectation varied. Male respondents reported that marriage generally entailed an expectation of higher behavioral standards, such as fidelity and increased romantic attention to one’s spouse, compared to cohabitation. Men and women felt that marriage could keep a relationship together through difficult times, such as infidelity. Men said that being married meant that even if they were unfaithful to their wives, their wives would still be obligated to stay with them and less able to leave them. Women expressed that being married provided legitimacy and a claim in the relationship as the “original” wife, describing marriage as a “strong defense against the other woman”, demonstrating a fear of infidelity and relationship instability that shapes relationship decisions and norms. Additionally, several cohabiting men suggested that marriage could be an important legal link to their children, and in case of conflict with the mother, they could not be denied access to their children.

### Social expectations and tradition

#### Cohabitation and childbearing

A very frequently discussed reason to cohabit was pregnancy. Respondents explained that marriage used to be the required response to a nonmarital pregnancy (i.e., all respondents in one focus group of married women reported that they married because they were pregnant); most men and women believed that cohabitation was now an acceptable and often preferable option because cohabitation could allow a couple to co-parent without prematurely committing to marriage. When asked whether an unmarried couple should marry if the woman becomes pregnant, one female respondent said:

Respondent: “An attitude like that is old-fashioned. [For example,] when you get pregnant, you have to get married whether you like it or not… This practice is very archaic. Right now, we are in a modern generation already. If you get pregnant and you want to get married and you both love each other, so be it. But if you don’t love each other and you only did it [sex] out of impulsiveness—It’s okay if you don’t marry.”

-urban married female, age 40, high school educated.

Some respondents also said that having several children still did not necessitate marriage.

Moderator: “If the girl gets pregnant, should they marry?”

Respondent: “No, not really. I have five children with my live in partner… Up to this date our relationship gets stronger.”

-rural cohabiting male, age 41, high school educated.

While respondents said they would prefer for their children to have children within marriage, they did not want this at the expense of relationship quality.

Respondent: “But [if] it’s my daughter. I will ask her if she gets pregnant. If he loves the boy, she should marry the boy. But if she does not, I won’t let her marry the boy even if she is pregnant. I will raise and take care of my grandchild.”

-rural married male, age 37, college educated.

While respondents all acknowledged that childbearing in cohabitation is common and increasingly acceptable, there was some disagreement, mostly among men, on whether this was a positive development. Nonetheless, although some participants expressed preferences for how their own children should behave, they were generally hesitant to criticize other people’s family decisions, hinting at an ambiguity of social norms and tolerance of a wider range of biographies and family forms.

### Family and social expectations

Although respondents gave a variety of reasons why people cohabit and in what cases cohabitation is a good idea, they also said that people cohabited simply because it was now acceptable. One respondent spoke candidly about how social expectations and norms regarding sexual behavior have relaxed markedly:

Respondent: “Come on, [before], if you were seen walking off the street with someone after dark, people would have said that something ‘happened’ to you already. Nowadays, you see couples making out in public, no one cares. It's different now compared to before.”

-urban married female, age 45, college educated.

Regarding marriage and social and family expectations, respondents’ views were varied. Some cohabiting respondents, particularly men, reported being questioned about why they had not married, and some older married women respondents reported being forced or pressured into marriage by their parents because their parents believed that premarital sex had taken place. However, in neither case was family pressure viewed positively or as a good reason to marry. In contrast, respondents’ perspectives as parents themselves were decidedly more liberal. Respondents said that nowadays, “parents’ mentalities aren’t the same” as before. One urban married woman pointed out that pressuring a pregnant daughter to marry a “nobody”, such as an irresponsible or abusive man, would “just be a pity”. Another woman specifically said she did not want her daughter to marry when she became pregnant because the man was unsuitable.

Respondent: “If it were up to me, it's okay that she gets pregnant [and doesn’t marry] because, the guy does not have a job. My daughter just loves him. But, I tell my child, it's not me who has to deal with your husband… You make the decision. But as a parent, I didn't want her to get married in that kind of situation, the man doesn't have a job. Right, it's like [she's] at a disadvantage?”.

-urban married female, age 44, high school educated.

Respondents also raised the social expectation of a large wedding as an obstacle to marriage and a reason to cohabit instead. Respondents said that if someone in their community married in a church wedding, everyone in the community would expect to be invited. In Western contexts, a wedding may be viewed not as the beginning of a union but as a couple’s public expression of commitment to each other, made in the presence of family, friends, and society (Cherlin, 2004; Berrington et al. 2015). However, while respondents expressed that inviting everyone to a church wedding was expected, they did not mention public demonstrations of love, commitment, and family cohesiveness as the purpose of a big wedding. Instead, respondents emphasized God’s blessing as the main purpose of a church wedding, which is further explained in the next section.

### Religious values

Respondents did not mention religious prohibitions against nonmarital sex and nonmarital childbearing as reasons against cohabitation and for marriage. When prompted to discuss how religion influenced relationship decisions, respondents said that religion could actually hinder marriage, such as prohibition of intermarriage across religions. Respondents did not view the decision to cohabit or marry as a religiously motivated decision, and when prompted, replied that practical concerns were a higher priority than religious rules. For example, many women said that it was morally superior to use money to pay for your children’s education compared to a wedding. On a pragmatic level, respondents reported that it did not make sense to marry someone if you were unsure of the relationship, regardless of religious dogma. However, respondents felt strongly that a church wedding was superior to a civil wedding or cohabitation because it importantly provided the union approval and blessing from God, which was highly valued. Both married and cohabiting respondents reported that a civil ceremony meant you were married in the eyes of the law but a church ceremony meant you were married in the eyes of God.

Respondent: “Like, the one in court… that is still in the eyes of the people because an attorney is the one who [marries you]… right? In church, it's like God is your witness. That's the main thing of you getting married.”

- urban cohabiting male, age 20, education level unreported.

Respondent: “Ahh, for us, ah, it is important that there is a blessing from the church. One, for our beliefs, most of us here are Catholics eh. They say that when you have the blessing from church, grace and blessings will easily come to you.”

-rural married woman, age 40, high school educated.

## Discussion

Although we cannot ascertain temporal value change with cross-sectional data, our respondents repeatedly talked about changes over time in norms and values. Our analysis of the focus group results for individualistic or family-centric tendencies shows that relationship values may have changed dramatically toward an individualistic bent, but traditional values of family, children, and religion persist. Family and social expectations regarding marriage have weakened, with personal choice and fulfillment becoming priorities in relationship decisions, consistent with individualistic tendencies and the deinstitutionalization of marriage (Beck & Beck-Gernsheim, 1995; Cherlin, 2004). However, relationships, regardless of their legal status, remain inextricably intertwined with having children, and in this way, other views and norms remain family and child-centric. Having children, committing to parenting in a co-residential two-parent household, and ensuring children are strongly connected to and acknowledged by both maternal and paternal kin groups remain fundamental priorities. Children are not only crucial to achieving full personhood but also a key purpose of being in a relationship. In the next section, I discuss how the main findings relate to individualism and familism and the extant literature, as well as how increased tolerance has also engendered attitudes of ambivalence and life course flexibility.

### Individualism, familism, and ambivalence

Respondents expressed views of cohabitation and marriage often associated with individualism (Beck and Beck Gernsheim 1995)—such as acceptance of cohabiting families, the belief that cohabitation can be as committed as marriage, high valuation of personal fulfillment and relationship quality, and a tolerance of non-standard biographies. Although participants named several practical, emotional, and religious advantages of marriage, they also expressed that the decision to marry depends on personal circumstances and should not be rushed, even in the event of a nonmarital pregnancy. Marriage was neither ideologically rejected nor viewed as essential. This parallels studies of cohabitation in individualized Western contexts where the birth of a child, and legal and financial advantages might have roles in the immediate decision to marry, but are not valid reasons alone; instead, the quality of the relationship is the highest priority (Berghammer et al., 2014).

Earlier research in the Philippines found young men tended to have a more positive view of cohabitation than young women but were more concerned with the financial burden of hosting a wedding (Williams et al., 2007). In contrast, we found a tendency for women to describe the growing acceptance of cohabitation as a positive development, whereas men were more ambivalent. Cohabitation may allow more sexual freedom (i.e., sex outside marriage, a “non-marriage” option in response to pregnancy) for women than they have historically had, which may be valued by women who are traditionally held to stricter sexual standards of behavior. For men, cohabitation may erode some of the traditional authorities and privileges they had by virtue of being men. As previous studies have found, cohabitation can symbolize more independence for women who would particularly benefit from avoiding the traditional expectations and dependency marriage entails (Kiernan, 2004). For example, women may view marriage as requiring a stricter adherence to gendered division of domestic labour and prefer cohabitation because they believe it is a more modern, gender equitable arrangement (Reed, 2006). In the Philippine context specifically, men may also feel particularly burdened by the financial pressure of hosting a wedding, and view cohabitation less favorably because it outwardly signals the lack of economic resources to host a wedding (Williams et al., 2007). Nonetheless, several family-centric values persist, including the high value of children, the power of parenthood to bring a couple together, and the importance of family, as well as a high degree of religiosity. Benefits of cohabitation were discussed from a child-centric perspective, while personal legal and financial benefits were not mentioned at all. This differs starkly from qualitative studies of cohabitation and marriage in Western contexts where participants focus on what cohabitation or marriage mean for themselves and their romantic relationships, and not for the whole family (Perelli-Harris et al., 2014). Another contrast with Western contexts was the valuation of a church wedding as a couple’s recognition from God, not for family pressure or as a symbol of social commitment (Vignoli & Salvini, 2014).

Qualitative studies in Western countries disentangle cohabitation as a relationship and cohabitation as a context for childbearing because not all cohabiting couples have or plan to have children together (Hiekel & Castro-Martin, 2014), unlike this study where nearly all cohabiting participants had children with their partners and indeed their children were the very reason they were living with their partners. In contrast to other cohabitation studies where a shared surname between couples is viewed as an important symbol of social identity, love, and public commitment between a couple (Berrington et al. 2015), the shared surname most strongly prioritized by both male and female respondents in this study was between father and the child, emphasizing the importance of publicly acknowledging commitment to a child, versus between romantic partners. Still, respondents’ high valuation of children did not conflict with the view that the decision to cohabit or marry should be based on what was best for the couple, instead of following a normative family formation pathway. To meet the high valuation of both having children and a quality, fulfilling partnership, a more liberal attitude toward the chronology of life events is necessary, yielding a wider range of acceptable life trajectories.

The combination of child-centric values and acceptance of cohabitation in the Philippines has some similarity with other Asian countries where liberalized attitudes toward premarital sex have not undermined the importance of marriage, children, and a persistently strict ordering of life course events (Cheng, 2023; Song & Lai, 2022; Yu & Xie, 2021). In other Asian countries, “individualization without individualism” (Kyung-Sup & Min-Young, 2010) has led to late marriage and low fertility as risk reduction strategies to control the scope and duration of family life. Just as postponement and smaller family size may be life course strategies to engage in family life more manageably in other Asian contexts, childbearing within cohabitation and the reordering of life events in the Philippines could be a similar risk reduction strategy. The rapid emergence of cohabitation and nonmarital fertility—often framed as evidence of deinstitutionalization of the family in other contexts—may in fact be strategies to simultaneously entrench both traditional family preferences (i.e., having children and raising them in a two-parent household) and a personally fulfilling relationship. Since family life remains so important, reshaping a manageable trajectory is necessary, given competing external influences.

Similarly, because respondents repeatedly emphasized the impact of family policy change and divorce laws, another relevant perspective on cohabitation and nonmarital fertility may be “institutionalized individualization”, in which social structures, services, and policies prompt individuals to follow individualized living arrangements and lifestyles (Beck and Beck-Gernsheim 2002). Finally, respondents’ view of cohabitation as insurance against relationship problems may also be related to individualistic attitudes. Because an individualistic approach allows for personal preferences, it also promotes the view that family-related failures are personal failures, leading to high anxiety toward relationship instability or failures (Beck & Beck-Gernsheim, 1995; Bulcroft et al., 2000; Giddens, 1994).

### Shifting costs and benefits of cohabitation and marriage

A major point of discussions was how economic concerns influence family decisions. Cohabitation in the Philippines follows a negative educational gradient (Kuang et al. 2019) and respondents cited the high cost of marrying as a reason to cohabit, suggesting less privileged couples cohabit out of resource constraint, as in the U.S. and across Europe (Edin & Kefalas, 2005; McLanahan, 2004; Perelli-Harris et al., 2010), not ideological preference. However, the perceived cost of marriage must be considered within the context of changing norms, values, and policies in the Philippines, which have shifted the perceived cost/benefit ratios of both marriage and cohabitation.

First, the main financial reason to cohabit instead of marry overwhelmingly raised by respondents was the cost of a wedding reception, with very little discussion otherwise of overall financial stability. This differs from studies, for example in the U.S. and Thailand, where the perceived economic obstacles to marriage are not limited to wedding costs but also include achieving a certain level of economic affluence and stability prior to marriage (Edin & Kefalas, 2005; Esara, 2012). And in contrast to other studies of cohabitation in the U.S. (Reed, 2006), no one in the focus groups mentioned sharing household expenses, convenience, or housing issues as reasons to cohabit. Concern about paying for a wedding to the exclusion of other financial concerns raises questions such as, to what degree does “saving for a wedding” imply disinterest or unwillingness to marry and a more palatable way to express such feelings without inviting further questions? On the other hand, in a middle-income country like the Philippines that has not experienced a recent history of a strong welfare state, employment protections, or social security, it may be that feelings of economic insufficiency play out differently, with more focus on short-term concerns such as food security and immediate expenses. For example, respondents’ preference to spend money on their children instead of on a wedding may reflect the immediacy of their financial concerns and not devaluation or ambivalence about marriage.

Second, marriage may be perceived as too costly because it is now more optional, given the acceptability of cohabitation and the extension of cohabiting families’ rights. Respondents said the expense of weddings was why cohabitation had increased, but wedding celebrations have always required some outlay of resources. Moreover, respondents were widely aware of affordable and free options to legally marry. Perhaps, now that marriage is viewed as optional, spending money on a wedding seems like a luxury that is no longer worth the expense if one can avail of marriage’s main benefits through cohabitation. Conversely, more affordable options such as mass weddings may not truly be a realistic option for those who either experience significant social pressure or personally strongly value hosting a large wedding celebration.

Third, the decision to marry or cohabit often occurs in the context of pregnancy or a recent birth, when there are competing demands on attention and resources. Before the rise of nonmarital fertility in the Philippines, it was not necessary to choose between paying for a wedding or children’s expenses, since the wedding usually occurred before children were born. Now that partnering decisions are often made when there is already a child to consider, it follows that some may prefer to allocate resources to their children versus a wedding. As in other places where nonmarital fertility is common, childbearing in cohabitation may be evolving to become a public declaration of commitment similar to or in replacement of weddings (Berrington et al. 2015). This approach is in direct contrast to other Asian contexts where premarital conception or nonmarital birth would likely expedite marriage, not provide a reason to postpone it. Another contrast is the poor access to family planning and lack of legal abortion in the Philippines, relative to other Asian countries, which may also explain the different approaches to nonmarital conceptions. In the Philippines, unintended nonmarital pregnancy may be more likely to occur given poorer access to family planning, and also much more likely to be carried to term, given lack of legal access to abortion. The weakening social pressure to legitimize a nonmarital birth and the extension of rights to cohabiting families may have shifted the balance between the benefits and liabilities of marriage, making cohabitation more appealing.

### Future research

In contrast to previous Philippine studies, this study included respondents from a range of ages and perspectives. The cohabiters in the focus groups were also a heterogeneous group, some of whom had recently partnered and others who had cohabited for over ten years with several children, suggesting that cohabitation does not have a single meaning, as reflected in the range of responses given by participants. Since the study data have been collected, further change may have occurred in the Philippines, precipitated by events such as the introduction of legislation to legalize divorce, the growth of social media and online dating apps. Future qualitative research could examine these new changes. Moreover, while focus groups comprised of a mix of ages and education levels are useful for creating the normative context in which values and social norms can emerge and be discussed, challenged, and reaffirmed, future work could more specifically examine attitudes by age group and education level to facilitate group discussion among people of similar demographic characteristics and more specifically test extant theories. Moreover, a wider range of locations could be explored in the future, including Muslim majority regions where family norms and religiosity may be different.

## Contributions

Using the case of the Philippines, this paper shows new perspectives on cohabitation and marriage reflecting both individualistic and family-centric orientations. Respondents’ perception of cohabitation as a common and acceptable arrangement for having and raising children contrasts strikingly with Philippine studies conducted nearly two decades ago when cohabitation was perceived disapprovingly and solely as a prelude to marriage for young people, hinting at rapid change of how cohabitation is viewed and the role of marriage. This study suggests the meaning of cohabitation may have rapidly shifted to become more like marriage, which to date, contrasts dramatically with other Asian contexts. Most strikingly, cohabitation is not only prevalent in a family-centric culture such as the Philippines but is in part motivated precisely by family and child-centric values. Although cohabitation behaviors and norms have changed profoundly, the focus on and high valuation of family in the Philippines have remained consistent. In other words, people’s partnering practices may have reshaped not to accommodate an entirely new set of values but to preserve the same values while adapting to new policy circumstances, relationship standards, and other realities.

Also notable is that value change and innovative family behaviors were observed among the non-elite. Focus group respondents primarily represented medium levels of education, not the highly educated forerunners of social change referred to by Second Demographic Transition theory who pioneer new family behaviors that gradually trickle down to other classes. In the Philippines, value change and widespread behavior change appear to be happening rapidly and simultaneously, without a changing socioeconomic gradient (Kuang et al. 2019). Although cross-sectional qualitative data are insufficient for identifying value change, it is noteworthy that respondents overwhelmingly reported that social norms had changed recently, with many citing new family policies and modern, liberalized attitudes as relevant drivers.

This study offers a new perspective that rethinks family behaviors as individualistic and liberal or family-centric and conservative by exploring the extent to which the same behavior can be re-claimed to address both individual needs and family values. In doing so, this paper challenges the notion that family norms and behaviors must either serve individual fulfillment or traditional, family-centric values. While emerging work in Asia examines a number of different family behaviors as evidence of continuity and change (i.e., persistence of marriage alongside rise in premarital pregnancy, respectively), this paper demonstrates how one specific behavior, cohabitation, reflects both continuity (familism) and change (individualism) at the same time. Contrary to the notion that only individualized societies prioritize higher order needs over prescribed family behaviors, the Philippine case demonstrates that societies with strong family ties can also highly value personal emotional fulfillment, adapt behaviors accordingly, and evolve to accept a wide range of life course trajectories. This contributes to the qualitative literature on the meanings and concepts of cohabitation, a literature which is currently dominated by Western experiences and ideas, even as cohabitation has increased across the world.

## Data Availability

The qualitative focus group data used in this study were collected by the author and a team at the University of the Philippines and are not publicly available due to the nature of confidentiality agreements with study participants.

## Footnotes

1 5,000 pesos in 2016 was approximately 93 euros
